# Supplementary material for: Identification and Functional Characterization of Oxidosqualene Cyclases from Medicinal Plant Hoodia gordonii
Source: Plants (Basel). 2024 Jan 14;13(2):231. doi: 10.3390/plants13020231 (PMC10818575; doi:10.3390/plants13020231)
Supplement: Supplementary file 1 [file plants-13-00231-s001.zip › plants-2808266-supplementary.pdf]

Identification and Functional Characterization of Oxidosqualene Cyclases from Medicinal Plant *Hoodia gordonii*

Iffat Parveen [1,*], Mei Wang [3], Joseph Lee [1], Jianping Zhao [1], Yingjie Zhu [2], Amar G. Chittiboyina [1], Ikhlas A. Khan [1,4] and Zhiqiang Pan [3,*]

Supplementary Information

Figure S1: Graphical representation of *Hoodia gordonii* **Hg**OSCs conserved domains.

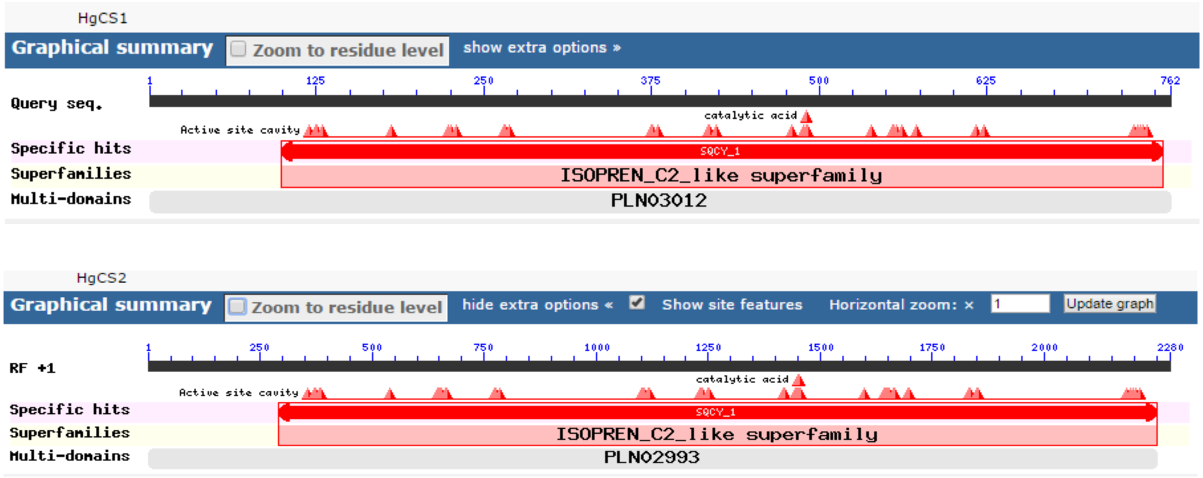

Figure S2. Amino acid alignments of HgOSC1 and HgOSC2 with oxidosqualene cyclases from plants. Four motifs rich motifs (Motif A-D), necessary for the binding of cationic cofactors are shown as boxes.

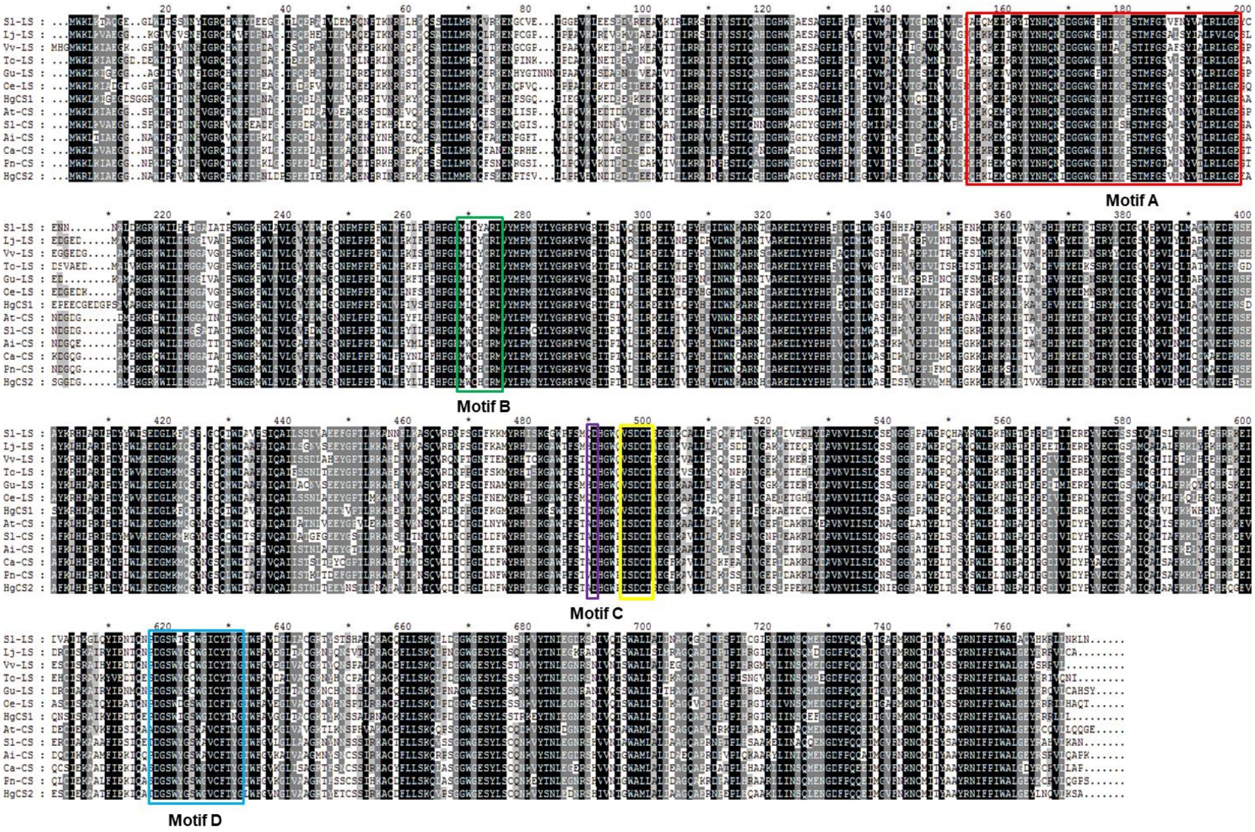

Figure S3: GC-MS mass spectrum of Lupeol

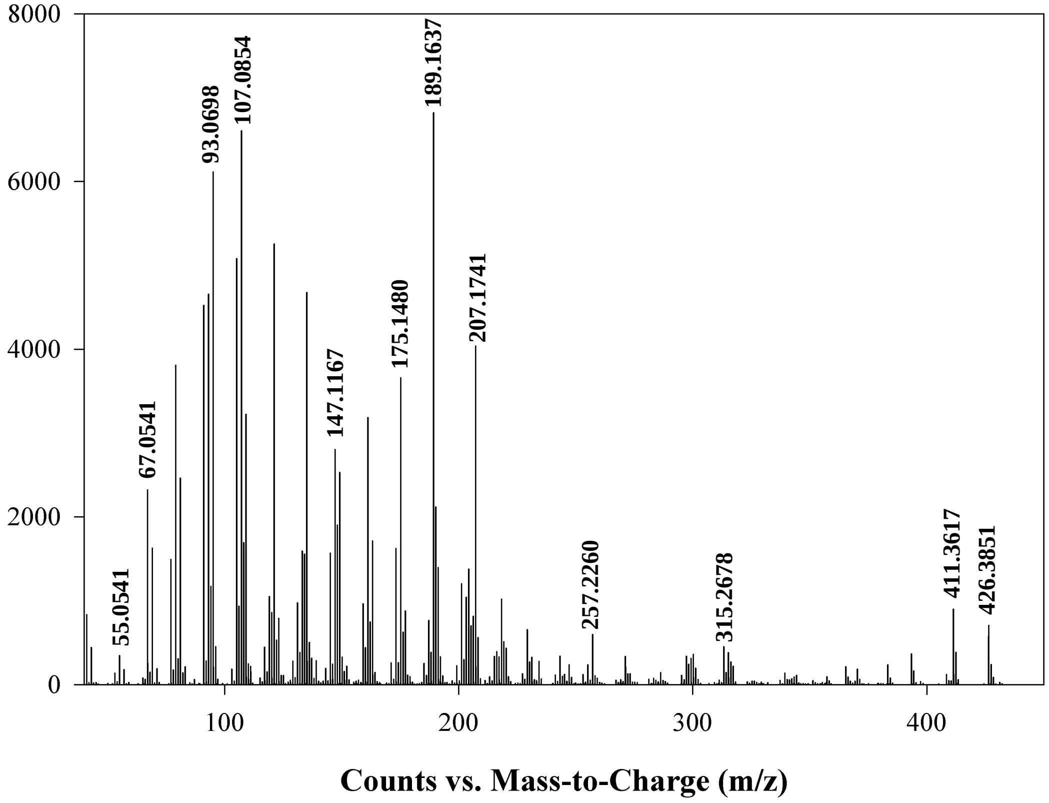

Figure S4. Thin-layer chromatography (TLC) and nuclear magnetic resonance (NMR) spectroscopy analyses. A, Compound extracts were separated on TLC (HG: hexane extract from yeast expressing HgOSC1; R: lupeol reference standard; V: hexane extract from yeast harboring vector as control). B and C, The ^{1}H (B) and ^{13}C (C) NMR spectra showing signals of the HgOSC1 product and the lupeol standard. The major signals of the ^{1}H and ^{13}C NMR spectra of the HgOSC1 product (lower panels of B and C) are comparable to those of the lupeol standard (upper panel of B and C).

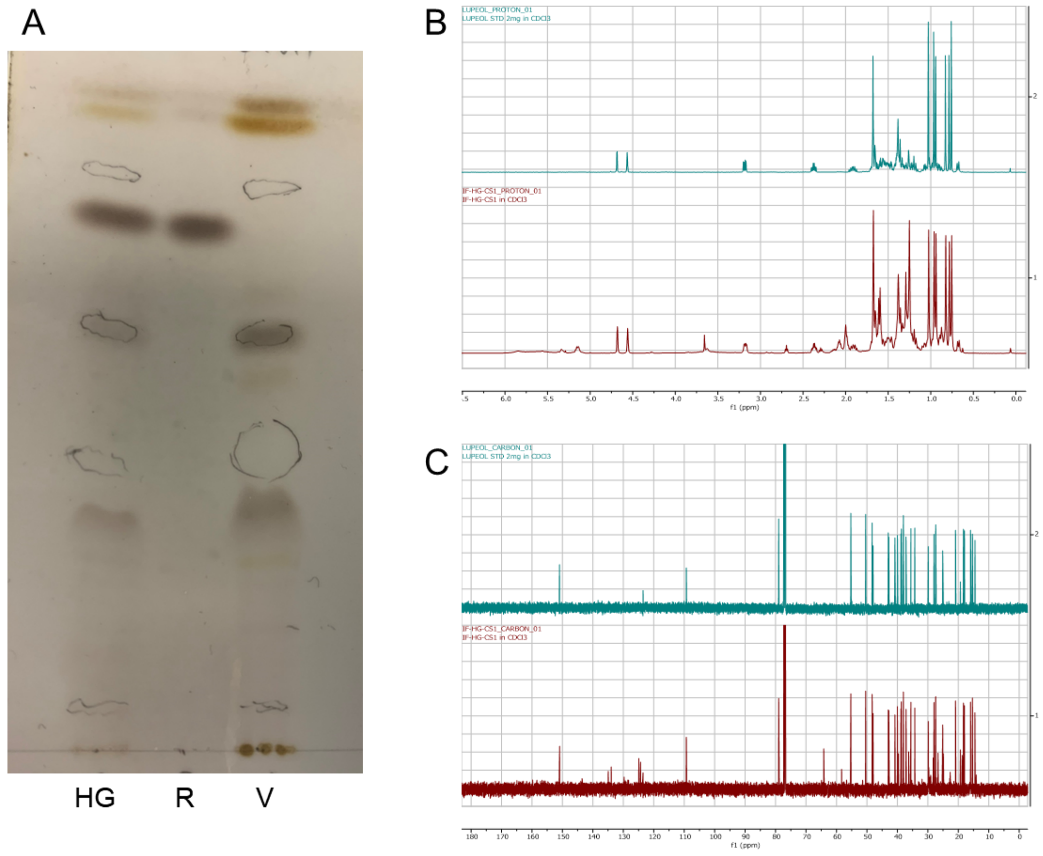

Figure S5. DNA sequence of *HgOSC2* synthesized for optimization of codon usage for expression in yeast.

ATGTGGAGATTGAAAATTGCTGAAGGTGGTAACGCATGGTTGAGAACTGTTAACAACTATGTTGGTAGACAATTCTGGGAATTCGATCCAAATT
TGGATCCATCTCCAGAAGAAATCGAAGAAATCGAAAAAGCTAGAGAAAACTTCAGAATTAATAGATTCGAAAAGAAACATTCAGCAGATTTGA
TGATGAGAATCCAATTTTCTAAGGAAAACCCAACTTCAGTTTTGTTGCCAAGAGTTGAAGTTAACGATATCGAAGATTTGACAGAAGAAAATGT
TACTTTGACATTGAAGAGAGCTATTAATTTCTATTCTACTTTACAAGGTCATGATGGTCATTGGGCTGGTGACTACGGTGGTCCAATGTTTTTGTT
ACCAGGTTTAGTTATTGCTTTGTCTATTACAGGTGCTTTGAACGCAGTTTTGTCAAAGCAACATAAGTTGGAAATGTGTAGATATTTGTACAACC
ATCAAAACATCGATGGTGGTTGGGGTTTGCATATTGAAGGTCCATCTACTATGTTCGGTTCAGTTTTAAACTACGTTACATTGAGATTGTTAGGTG
AAGAAGCTTCTGGTGGTGACGGTGCAATGGAAAAAGGTAGAAAATGGATTTTGGATCATGGTGGTGCTACTGCAATTACATCTTGGGGTAAAA
TGTGGTTATCAGTTTTGGGTGCTTATGAATGGTCTGGTAATAATCCATTACCACCAGAAATTTGGTTGTTACCATACTTGTTACCATTTCATCCAGG
TAGAATGTGGTGTCATTGTAGAATGGTTTATTTGCCAATGTCTTATTTGTACGGTAAAAGATTCGTTGGTCCAATCACTCCAACAATCTTGTCATTG
AGAAGAGAATTGTACACAGTTCCATACCATGAAGTTGATTGGAATAAGGCTAGAAACCATTGTGCAAAGGAAGATTTGTACTACCCACATCCAT
TGATCCAAGATATCTTGTGGGCTTCTTTGGATTCATTTGTTGAACCAGTTATGATGCATTGGCCAGGTAAAAAGTTGAGAGAAAAGGCATTGAG
AACTGTTATGGAACATATCCATTACGAAGATGAAAACACAAGATACATCTGTATCGGTCCAGTTAATAAGGTTTTGAACATGTTGTGTTGTTGGG
TTGAAGATCCAACTTCTGAAGCTTTTAAATTGCATATCCCAAGAGTTCATGATTATTTGTGGTTGGCAGAAGATGGTATGAAAATGCAAGGTTAC
AATGGTTCACAATTATGGGATACAGCTTTCGCAGTTCAAGCTATCATCTCTACTAATTTGACAGAAGAATATAATTCAACTTTGAGAAACGCTCAT
GCATACATTAAGAATACACAAGTTCAAGATGATTGTCCTGGTGACTTGGATTTCTGGTACAGACATATCTCTAAGGGTGCTTGGCCATTTTCAACT
GCAGATCATGGTTGGCCAATCTCTGATTGTACATCAGAAGGTTTGAAGGCTGTTTTGTTGTTGTCTAAGTTGTCACCAGAATTGGTTGGTGAATC
TTTGCCACCAGAAAAGTTGTACGATGCTGTTAACGTTATCTTATCATTGCAAAATATTGAAGGTGGTTTCGCAACTTACGAATTGACAAGATCTTA
CTCATGGTTAGAATTGATTAATCCAGCTGAAACTTTCGGTGACATCGTTATCGATTACCCATACGTTGAATGTACATCTGCTGCAATTCAAGCATTG
GCTGCTTTTAAGAAATTGTACCCAAGACATAGACAAGGTGAAGTTGAATCATGTATCGAAAAGGCTGCAACTTTTATTGAAAAGATTCAAGCTA
CAGATGGTTCTTGGTATGGTTCATGGGGTGTTTGTTTTACTTACGGTTTGTGGTTTGGTGTTAATGGTTTAGTTGCTGCAGGTAGAACTTACGAA
ACATGTTCTTCAATCAGAAAGGCATGTGATTTCTTGTTGTCTAAACAAGTTCCATCAGGTGGTTGGGGTGAATCTTATTTGTCATGTCAAAATAA
GGTTTACTCTAATTTGGAAGATAACAGATCACATATCGTTAATACTGGTTGGGCTATGTTAGCATTGATTGCTGCAGGTCAAGCTGAAAGAAATC
CAGAACCATTGCATCAAGCTGCAAAGTTGTTGATTAATTCTCAATTAGAAAATGGTGACTTTCCACAACAAGAAATTATGGGTGTTTTTAATAAG
AACTGTATGATCACATACGCTGCATACAGAAACATCTTCCCAATCTGGGCTTTGGGTGAATATTTGAATCAAGTTTTAAAATCTGCATAA

Figure S6: The corresponding mass spectra of (a) Squalene; (b) Cycloartenol; (c) Ergosterol and (d) Cycloartenol acetate from GC-MS analysis.

(a)

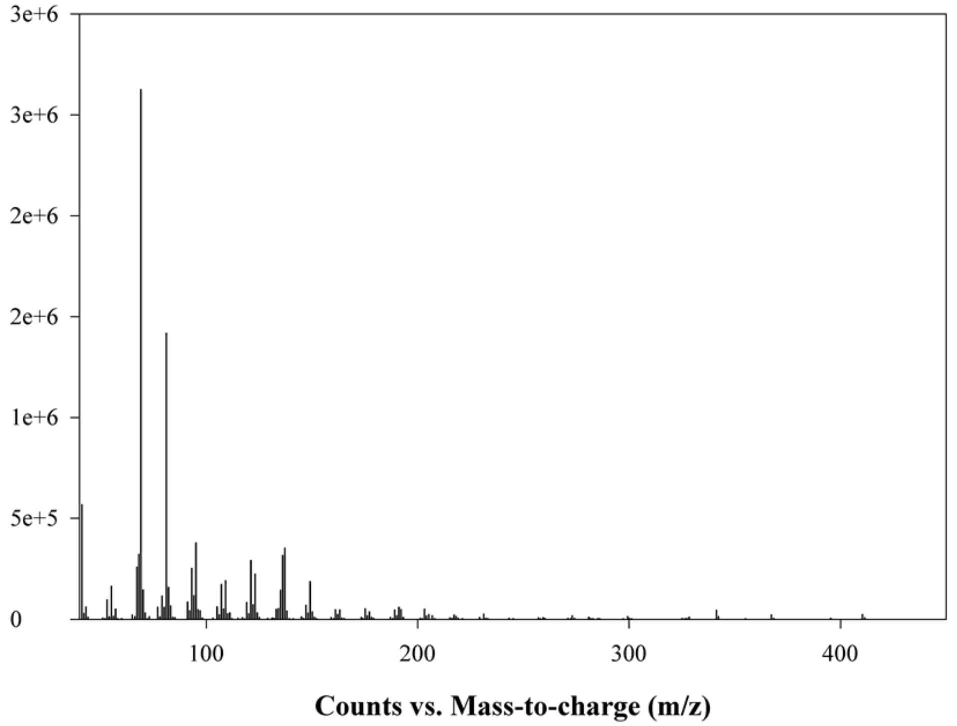

(b)

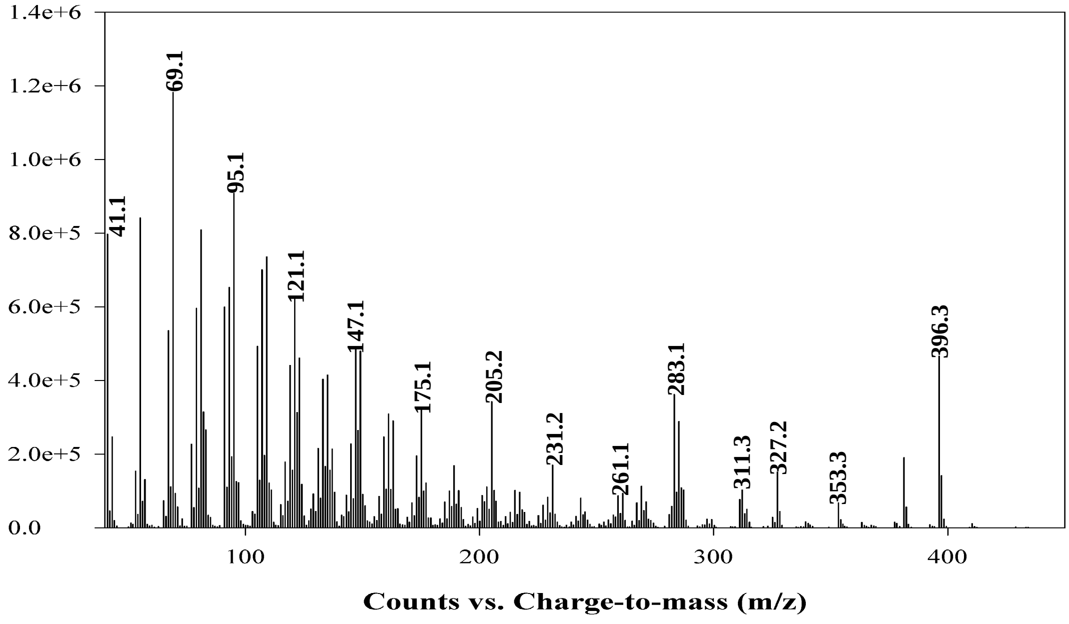

(c

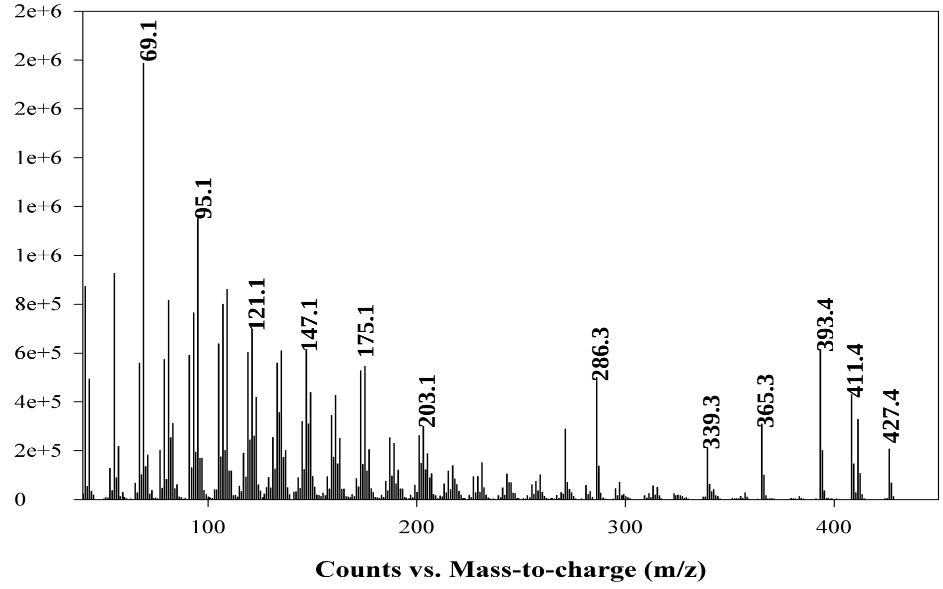
2e+6
2e+6
2e+6
1e+6
1e+6
1e+6
8e+5
6e+5
4e+5
2e+5
0
69.1
95.1
121.1
147.1
175.1
203.1
286.3
339.3
365.3
393.4
411.4
427.4
100
200
300
400
Counts vs. Mass-to-charge (m/z)

(d

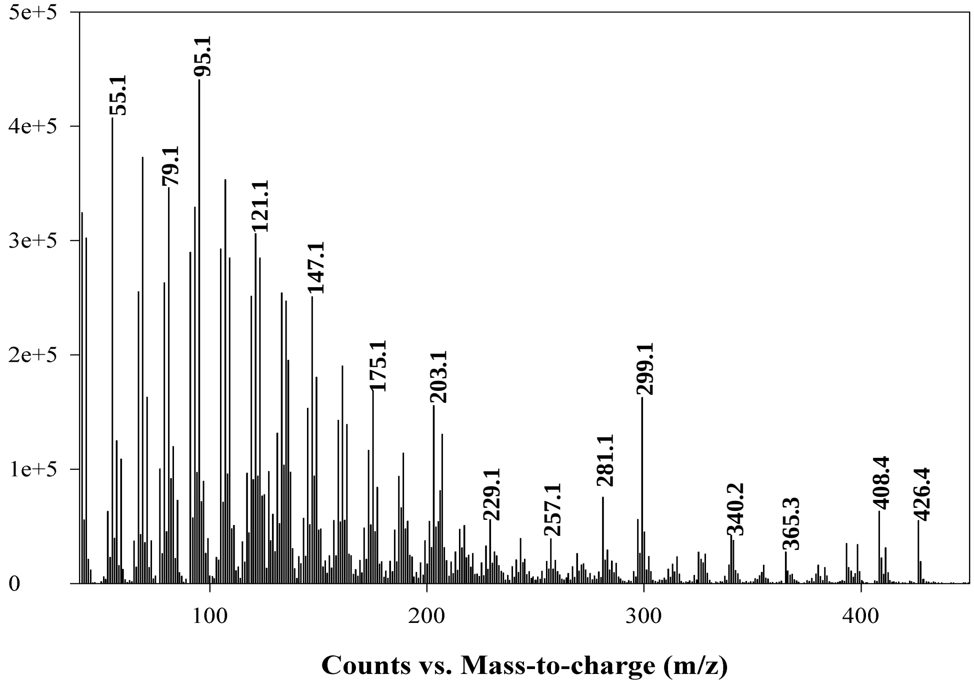
5e+5
4e+5
3e+5
2e+5
1e+5
0
55.1
79.1
95.1
121.1
147.1
175.1
203.1
229.1
257.1
281.1
299.1
340.2
365.3
408.4
426.4
100
200
300
400
Counts vs. Mass-to-charge (m/z)

Table S1: Primers used for 5'- and 3' rapid amplification of cDNA ends (RACE).

Primer Code	Locus	Sequence (5'-3')
P711	hga_locus_5882_iso_1_len_575 (F)	TCCTGATGATGGTGATGGTGCTGTT
P712	hga_locus_5882_iso_1_len_575 (Rev)	AGGACATTGGCATATAGGTGGTTCGAC
P713	hga_locus_5300_iso_4_len_427 F	TCTACCTCCTCTGGTGATGGCGTTGT
P714	hga_locus_5300_iso_4_len_427 R	CCACATTCTTCAGGCTCTGCTGCTTCT
P715	hga_locus_3813_iso_1_len_390 F	TGTTGGACGCATAACTGAGCTTGT
P716	hga_locus_3813_iso_1_len_390 R	AGCATATCTTGAGGTAAAGGATGAGGGT
P717	hga_locus_11949_iso_1_len_446 F	AAGCCCTTCGTACTGTGATGGAACA
P718	hga_locus_11949_iso_1_len_446 R	CTCAGTCAGGTTTGTCGAAATAATCGCC
P719	hga_locus_2117_iso_1_len_589 F	CTGAAGATAGCGGAGGGAGGGAA
P720	hga_locus_2117_iso_1_len_589 R	TCGTGGTAACAATACACTGGTTGGG
P721	hga_locus_5625_iso_3_len_1014 F	AGGCATCGTCAAGGAGAGGTGGAAAG
P722	hga_locus_5625_iso_3_len_1014 R	GTCCAGCAGCAATGAGAGCCAGCA
P723	hga_locus_5748_iso_1_len_268 F	TTGAAGATACACAGGAACCAGATGGC
P724	hga_locus_5748_iso_1_len_268 R	TACCACAGGCAGTCAATCCTCCTAC
